# Facile Synthesis of Island-like ZrO_2_-VO_2_ Composite Films with Enhanced Thermochromic Performance for Smart Windows

**DOI:** 10.3390/ma16010273

**Published:** 2022-12-28

**Authors:** Jiahao Wu, Zhe Wang, Bin Li, Baoshun Liu, Xiujian Zhao, Gen Tang, Dawen Zeng, Shouqin Tian

**Affiliations:** 1 State Key Laboratory of Silicate Materials for Architectures, Wuhan University of Technology (WUT), No. 122, Luoshi Road, Wuhan 430070, China; 2 Science and Technology on Aerospace Chemical Power Laboratory, Hubei Institute of Aerospace Chemotechnology, Xiangyang 441003, China; 3 State Key Laboratory of Materials Processing and Die & Mould Technology, Huazhong University of Science and Technology, No. 1037, Luoyu Road, Wuhan 430074, China

**Keywords:** magnetron sputtering, VO_2_-ZrO_2_, acid-solution process, thermochromic properties

## Abstract

VO_2_-based film, as a very promising thermochromic material for smart windows, has attracted extensive attention but has not been widely applied because it is difficult to simultaneously improve in terms of both solar-modulation efficiency (Δ*T*_sol_) and visible transmittance (*T*_lum_) when made using the magnetron-sputtering method, and it has poor durability when made using the wet chemical method. Herein, island-like ZrO_2_-VO_2_ composite films with improved thermochromic performance (Δ*T*_sol_: 12.6%, *T*_lum_: 45.0%) were created using a simple approach combining a dual magnetron-sputtering and acid-solution procedure. The film’s Δ*T*_sol_ and *T*_lum_ values were increased initially and subsequently declined as the sputtering power of the ZrO_2_ target was raised from 30 W to 120 W. Δ*T*_sol_ achieved its maximum of 12.6% at 60 W, and *T*_lum_ reached its maximum of 51.1% at 90 W. This is likely the result of the interaction of two opposing effects: Some VO_2_ nanocrystals in the composite film were isolated by a few ZrO_2_ grains, and some pores could utilize their surface-plasmon-resonance effect at high temperature to absorb some near-infrared light for an enhanced Δ*T*_sol_ and *T*_lum_. More ZrO_2_ grains means fewer VO_2_ grains in the composite film and increased film thickness, which also results in a decrease in Δ*T*_sol_ and *T*_lum_. As a result, this work may offer a facile strategy to prepare VO_2_-based films with high thermochromic performance and promote their application in smart windows.

## 1. Introduction

Thermochromic windows are considered a promising way to reduce building energy consumption significantly due to their simple structure, good solar modulation, and zero-energy input characteristics [1]. Among these thermochromic films, VO_2_ films have attracted much attention because they can modulate near-infrared (NIR) transmittance via their reversible and ultra-fast transition between monoclinic phase (M phase) and rutile phase (R phase) at about 68 °C [1]. In this sense, VO_2_ thermochromic smart windows can respond to changes in the surrounding temperature and then automatically adjust the amount of solar radiation entering indoors. The smart windows can block NIR from entering the room when the temperature is high, thus reducing energy consumption caused by air conditioning, and they can allow NIR to enter indoors when the temperature is low in winter, raising the indoor temperature and thus reducing energy consumption caused by warming. Therefore, VO_2_ films have emerged as a promising material for the upcoming generation of smart-window coatings due to the above advantages. In fact, VO_2_ films are not widely applied in buildings, mainly because of undesirable modulation efficiency (Δ*T*_sol_), the inherent low visible luminous transmittance *T*_lum_, high phase-transition temperature (*T*_c_), and lackluster durability [2,3,4]. In particular, Δ*T*_sol_ and *T*_lum_ are especially difficult to improve simultaneously.

To solve the above issues, numerous techniques have been employed, such as doping, a multilayer-film structure, a core–shell structure, interfacial tension, etc. Many metal oxides such as TiO_2_, ZnO, WO_3_, and ZrO_2_ have been introduced into VO_2_-based films as layers to form multilayer VO_2_-based films for enhanced thermochromic performance. ZrO_2_ is one of the metal oxides that may be utilized as a protective layer and anti-reflection layer for VO_2_ films since it is relatively stable and has a good refractive index. ZrO_2_/VO_2_/ZrO_2_ three-layer films were prepared by pulsed-laser deposition and showed enhanced visible transmittance while Δ*T*_sol_ remained at only 8% [5]. Additionally, Zr doping has a significant impact on the thermochromic performance of VO_2_-based films [6,7,8]. For instance, ZrW_2_O_8_/VO_2_/ZrW_2_O_8_ thin films exhibited good light transmittances of 71.9% compared to 39.8% of intrinsic VO_2_ thin films [6]. W/Zr co-doped VO_2_ nanoparticles synthesized by a hydrothermal method showed excellent thermochromic properties with *T*_c_, *T*_lum_, and Δ*T*_sol_ of 46.9 °C, 60.7%, and 10.6%, respectively [8]. In this regard, Zr doping or the addition of ZrO_2_ in VO_2_-based films would result in an evident improvement in the thermochromic performance. However, these VO_2_-based films do not exhibit good stability [9,10,11,12,13]. Hence, dual magnetron sputtering is employed to prepare ZrO_2_-VO_2_ composite films because the films obtained by sputtering usually have strong adhesion and thus exhibit good stability.

Unfortunately, the VO_2_-based films prepared by magnetron sputtering are dense and absorb more visible light, leading to a low visible transmittance. In order to improve the visible transmittance, a solution process is usually employed to fabricate porous VO_2_ films [14,15]. The porous VO_2_ films exhibit enhanced visible transmittance due to the increased porosity and good solar-modulation efficiency because of strong localized surface-plasmon-resonance (LSPR) effects at high temperatures caused by small isolated VO_2_ grains [16,17,18]. Therefore, the method combining magnetron sputtering and a solution process is a promising way to prepare VO_2_-based film with enhanced thermochromic performance. However, the method was not employed to synthesize ZrO_2_-VO_2_ composite films because ZrO_2_ grains showed better solution resistance than VO_2_. In this work, island-like ZrO_2_-VO_2_ composite films on glass were created using a simple technique combining dual magnetron sputtering and an acid-solution process. The obtained ZrO_2_-VO_2_ composite films showed enhanced thermochromic performance compared with pure VO_2_ films. The effect of ZrO_2_ content on the thermochromic properties was investigated in detail to account for the enhancement in thermochromic performance.

## 2. Materials and Methods

**Figure 1:**
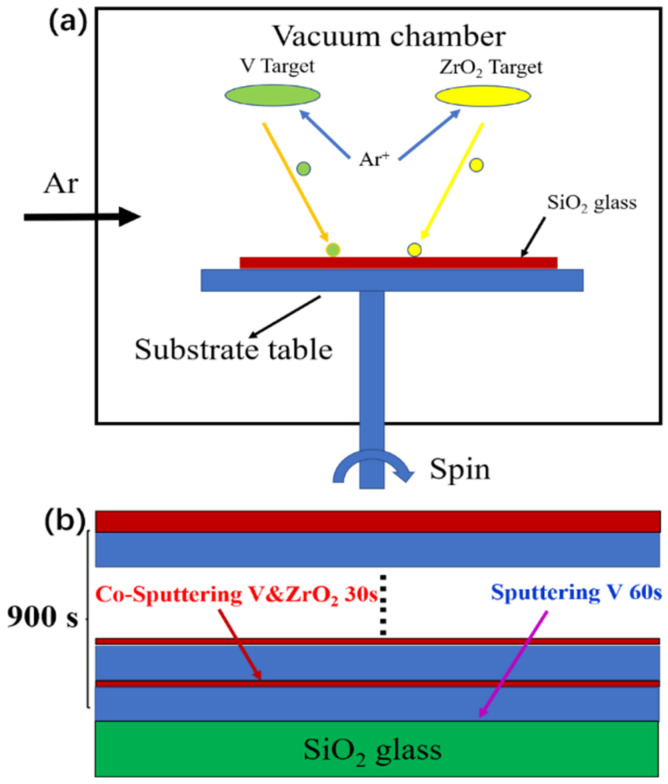
(**a**) Magnetron-sputtering diagram and (**b**) diagram for the ZrO_2_-V composite-film-preparation process.

Without additional purification, all chemicals were utilized after being acquired from Sinopharm Chemical Reagent Co., Ltd. In this experiment, Nanchang National Materials Technology Co., Ltd.’s V target (99.95% purity) and ZrO_2_ target (99.99% purity) were employed. The magnetron-sputtering equipment used in the experiment was JPD-500. The size of the vacuum chamber was Φ500 × H420 mm and the size of rotating substrate table was Φ150 mm. Figure 1a displays magnetron-sputtering diagram. Before sputtering, cooling water was turned on. It was ensured that the vacuum chamber was evacuated to 3.0 × 10^−3^ Pa. At this time, argon with a purity of 99.99% was introduced into the reaction chamber and the flow of argon was adjusted to 200 sccm. First, ZrO_2_-V composite films were deposited through direct-current magnetron sputtering of V targets at a power of 90 W and radio-frequency magnetron sputtering of ZrO_2_ targets simultaneously at various power. The total continuous sputtering duration of the V target was 15 min, and the zirconia target was intermittently sputtered 9 times for 30 s each time. The sputtering pressure was 0.4 Pa. Figure 1b depicts the comprehensive manufacturing procedure of ZrO_2_-V composite films. Finally, ZrO_2_-VO_2_ composite films were obtained through post-annealing the ZrO_2_-V composite films in a tube furnace. Specifically, the films were specifically placed in a tube furnace with an air pressure of 1000 Pa. the temperature was ramped up to 450 °C and held for 1 h at a rate of 5 °C/min, followed by a natural cooling process. For comparison, ZrO_2_-VO_2_ composite films with different sputtering power of ZrO_2_ were denoted as sample ZrO_2_-30 W, ZrO_2_-60 W, ZrO_2_-90 W, and ZrO_2_-120 W.

ZrO_2_-30 W, ZrO_2_-60 W, ZrO_2_-90 W, and ZrO_2_-120 W were placed in a PTFE etching flower basket and each was firstly subjected to a 5 s treatment in hydrochloric acid at a molar concentration of 5.6 mol/L, noting that there was no obvious change in optical properties after the acid-solution process. The acid-solution-process time was therefore increased to 20 s. Immediately after corrosion, the films were taken out of the acid solution and ultrasonically cleaned for one minute in deionized water and anhydrous ethanol. The obtained VO_2_-based films were dried with N_2_ and labelled as ZrO_2_-30 W-acid, ZrO_2_-60 W-acid, ZrO_2_-90 W-acid, and ZrO_2_-120 W-acid. The preparation conditions of pure VO_2_ film and ZrO_2_-doped VO_2_ film are shown in Table 1.

The crystal structure of the film was characterized on an Empyrean diffractometer using grazing angle X-ray diffraction (GAXRD) measurement (Cu Kα, λ = 0.154178 nm, generated at 4 kW output power). The morphology and element distribution of the film were characterized by a field-emission scanning-electron microscope (SEM, JSM-5610LV, Tokyo, Japan). The valence and composition of elements of the film were determined by X-ray photoelectron spectroscopy (XPS, ESCALAB 250Xi/ESCALAB 250Xi. Thermo Fisher, Waltham, MA, USA). To evaluate the optical properties of composite films, the solar transmittance of the film in the range of 300–2500 nm at 20 °C and 90 °C was analyzed by an ultraviolet-visible near-infrared spectrophotometer (UV-3600, Shimadzu Corporation, Kyoto, Japan). The integrals *T*_lum_ and Δ*T*_sol_ were calculated with the following Formulas (1) and (2) [1,2,3,19,20,21,22,23,24,25].

(1) $$T_{\mathrm{lum}}=\int_{380}^{780}\nu_{m}\left(\lambda \right)T\left(\lambda \right)d\lambda /\int_{380}^{780}\nu_{m}\left(\lambda \right)d\lambda$$

(2) $$T_{\mathrm{sol}}=\int_{300}^{2500}\varphi_{\mathrm{sol}}\left(\lambda \right)T\left(\lambda \right)d\lambda /\int_{300}^{2500}\varphi_{\mathrm{sol}}\left(\lambda \right)d\lambda$$

The Δ*T*_sol_ was calculated with Formula (3).

(3) $$\Delta T_{sol}=T_{sol}\left(20{}^{\circ}C\right)-T_{sol}\left(90{}^{\circ}C\right)$$

where *T*(λ) is the transmittance, λ is the wavelength of the incident light, *ν*_m_(λ) denotes the spectral sensitivity of the light to the human eye, and *φ*_sol_(λ) represents the irradiance spectrum of the sunlight at an atmospheric mass of 1.5 (corresponding to the sun from the horizon 37°) [1,2,3,26,27,28,29].

## 3. Results and Discussion

### 3.1. Structures of the Films before and after Acid-Solution Treatment

**Figure 2:**
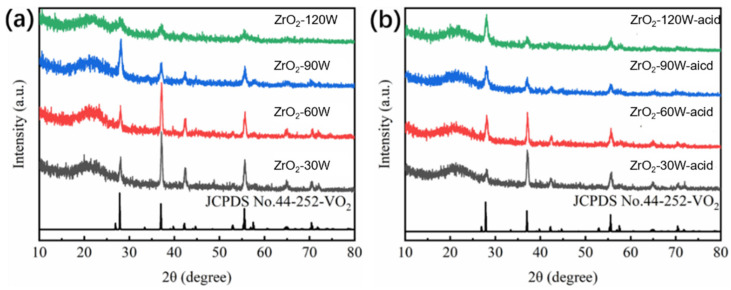
XRD patterns of (**a**) sample ZrO_2_-30 W, ZrO_2_-60 W, ZrO_2_-90 W, and ZrO_2_-120 W and (**b**) sample ZrO_2_-30 W-acid, ZrO_2_-60 W-acid, ZrO_2_-90 W-acid, and ZrO_2_-120 W-acid.

The XRD patterns of the samples before (ZrO_2_-30 W, ZrO_2_-60 W, ZrO_2_-90 W, ZrO_2_-120 W) and after (ZrO_2_-30 W-acid, ZrO_2_-60 W-acid, ZrO_2_-90 W-acid, ZrO_2_-120 W-acid) the acid-solution procedure are shown in Figure 2. It can be seen that every sample had strong diffraction peaks at 2θ = 27.9°, 37.1°, 42.4°, and 55.6°, which could be matched to VO_2_ (M) (JCPDS No.44-252, a = 5.753Å, b = 4.526Å, c = 5.383Å, space group: P2_1_/c, α = 90°, β = 122.6°). No further noticeable peaks were observed, with the exception of the amorphous peak of the glass substrate at 2θ = 20°.This indicates that VO_2_ in the film only exists in the form of M phase. In the XRD diagram, we did not find an obvious diffraction peak of ZrO_2_, which may have been due to the short sputtering time of ZrO_2_, and the content of ZrO_2_ prepared was lower than the XRD measurement limit. According to Goedicke’s experimental analysis, ZrO_2_ films prepared by magnetron sputtering are crystalline without annealing. There were obvious diffraction peaks at ZrO_2_ (111), ZrO_2_ (220), ZrO_2_ (211), and ZrO_2_ (220), and they grew preferentially with the increase in sputtering power. The successful preparation of ZrO_2_ was proven in the subsequent XPS test analysis [30,31,32,33]. 

From Figure 2a, it is clear that the VO_2_ film after the introduction of ZrO_2_ grains grew preferentially along the (020) crystal plane corresponding to 2θ = 37.1°. Additionally, the intensity of the peak at 2θ = 37.1° was progressively declined as sputtering power increased, showing the benefit of a proper ZrO_2_ concentration for the preferred growth of VO_2_ grains along the (020) crystal plane. Moreover, the film’s diffraction-peak intensity was decreased when the sputtering power reached 120 W. This is probably because the excess ZrO_2_ affected the crystallization of VO_2_. The intensity of the peak at 2θ = 37.1° was gradually decreased as the ZrO_2_ power increased after the acid-solution process, whereas the intensity of the peak at 2θ = 27.9° gradually increased, as shown in Figure 2b. This indicates that hydrochloric acid preferentially corrodes (020) facets of VO_2_ grains during the acid process [32,33,34,35].

**Figure 3:**
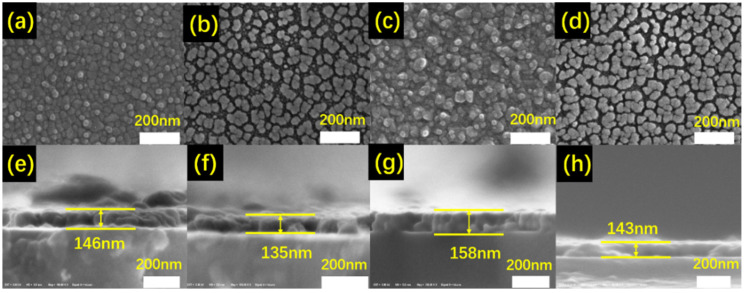
SEM images of the surface morphology of (**a**) sample ZrO_2_-60 W, (**b**) sample ZrO_2_-60 W-acid, (**c**) sample ZrO_2_-90 W, and (**d**) sample ZrO_2_-90 W-acid; cross sections of (**e**) sample ZrO_2_-60 W, (**f**) sample ZrO_2_-60 W-acid, (**g**) sample ZrO_2_-90 W, and (**h**) sample ZrO_2_-90 W-acid.

In order to further investigate the impact of the acid-solution process of the morphology evolution of the samples, SEM characterization was performed on samples ZrO_2_-60 W, ZrO_2_-60 W-acid, ZrO_2_-90 W, and ZrO_2_-90 W-acid, and the results are shown in Figure 3. It can be seen that the film’s grain size was comparatively homogeneous and became small after the acid-solution process. As the power increased, the film became rougher and gradually thicker before the acid-solution process. After the acid-solution process, voids gradually developed in the films to form numerous islands on the surface that resulted in a strong surface-plasmon-resonance (LSPR) effect and a higher Δ*T*_sol_, and the thickness of the films gradually decreased, which was extremely encouraging for achieving an increase in the films’ *T*_lum_ [36,37,38].

### 3.2. Thermochromic Properties of VO_2_-Based Films

**Figure 4:**
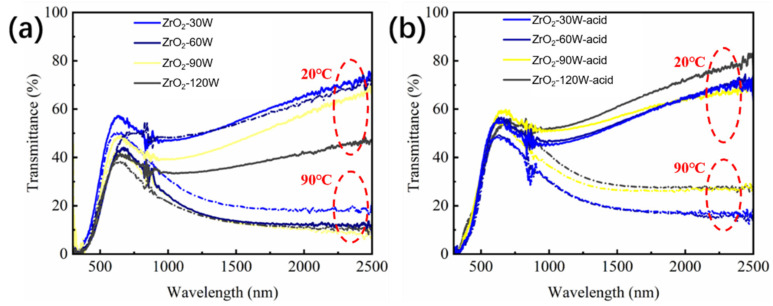
The transmittance of (**a**) samples ZrO_2_-30 W, ZrO_2_-60 W, ZrO_2_-90 W, and ZrO_2_-120 W, and (**b**) samples ZrO_2_-30 W-acid, ZrO_2_-60 W-acid, ZrO_2_-90 W-acid, and ZrO_2_-120 W-acid at 20 °C (line) and 90 °C (short point line), respectively.

**Figure 5:**
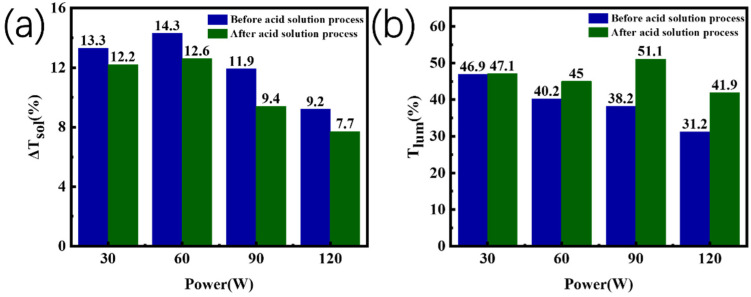
Comparison of (**a**) *∆T_sol_* and (**b**) *T_lum_* between ZrO_2_-VO_2_ composite films (before the acid-solution process) and island-like ZrO_2_-VO_2_ films (after the acid-solution process) with different sputtering powers of the ZrO_2_ target.

Figure 4a,b exhibit the solar transmittances of samples ZrO_2_-30 W, ZrO_2_-60 W, ZrO_2_-90 W, ZrO_2_-120 W, and samples ZrO_2_-30 W-acid, ZrO_2_-60 W-acid, ZrO_2_-90 W-acid, and ZrO_2_-120 W-acid, respectively, at 20 °C and 90 °C. Table 2 lists the films’ solar-modulation efficiency (Δ*T*_sol_) and luminous transmittance (*T*_lum_). When the sputtering power of the V target was 90 W, the pure VO_2_ film obtained by magnetron sputtering showed a Δ*T*_sol_ of 12.4% and *T*_lum_ of 28.2%. The VO_2_-based films’ *T*_lum_ increased dramatically when ZrO_2_ was introduced, reaching 46.9% when the ZrO_2_ target’s sputtering power was 30 W (ZrO_2_-30 W). This is because the introduction of ZrO_2_ decreased the refractive index of VO_2_-based films. Furthermore, the introduction of ZrO_2_ improved the crystallinity of the film, resulting in an increase in Δ*T*_sol_ to 13.3% at 30 W (ZrO_2_-30 W-acid) and 14.3% at 60 W. (ZrO_2_-60 W-acid). However, when the power exceeded 60 W (ZrO_2_-90 W and ZrO_2_-120 W), too much ZrO_2_ affected the oxidation and crystallization process of the V film, resulting in a decrease in *T*_lum_ and Δ*T*_sol_. After a 20 s treatment in hydrochloric acid at a molar concentration of 5.6 mol/L, the obtained films showed very good acid resistance. From Figure 5 and Table 2, the *T*_lum_ was increased after the acid-solution process, probably attributable to the reduction in film thickness and the generation of few pores in the film. The reduction of VO_2_ content in the film probably led to a decrease in Δ*T*_sol_. Consequently, it is unrealistic to enhance *T*_lum_ by extending acid-solution-processing time, which is in good agreement with previous works [32]. In particular, the ZrO_2_-VO_2_ obtained after the acid-solution process (ZrO_2_-90 W-acid) exhibited the highest *T*_lum_ of 51.1% while keeping a good Δ*T*_sol_ of 9.4%.

**Figure 6:**
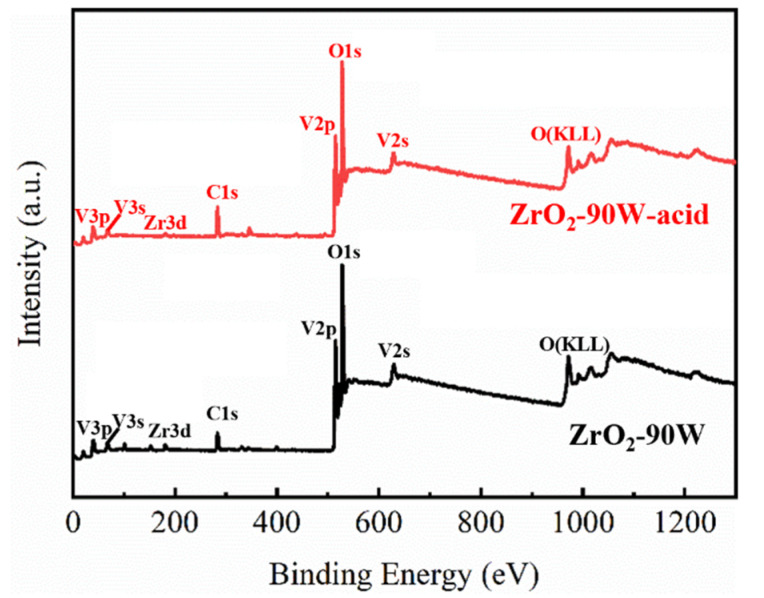
XPS survey spectrum of samples ZrO_2_-90 W and ZrO_2_-90 W-acid.

In order to investigate the effect of the acid-solution process on the composition of island-like ZrO_2_-VO_2_ composite film, XPS characterization was performed on samples ZrO_2_-90 W and ZrO_2_-90 W-acid, and the results are shown in Figure 6. It is evident that both films contained C, V, Zr, and O elements. The binding energies for O 1s, V 2p, Zr 3d, and C 1s, where the C contents came from adventitious carbon, were 530 eV, 515 eV, 180 eV, and 284.8 eV, respectively.

**Figure 7:**
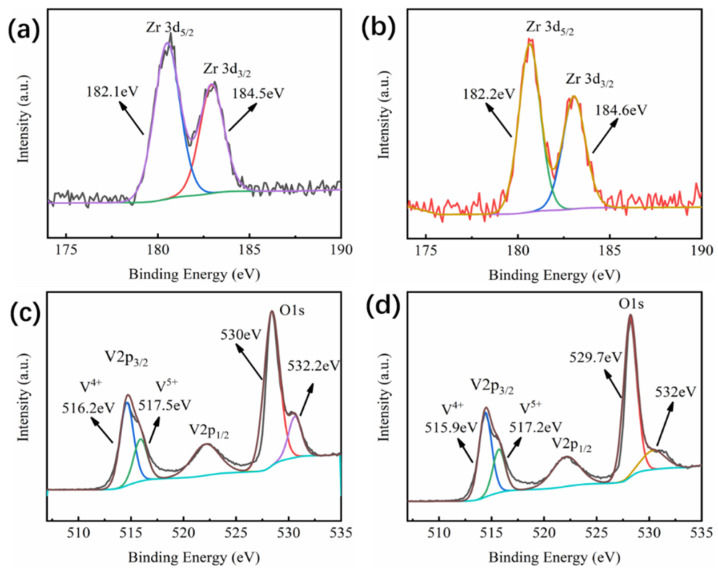
High-resolution XPS spectra for Zr 3d in (**a**) sample ZrO_2_-90 W and (**b**) sample ZrO_2_-90 W-acid, and V 2p and O 1s core levels in (**c**) sample ZrO_2_-90 W and (**d**) sample ZrO_2_-90 W-acid.

**Figure 8:**
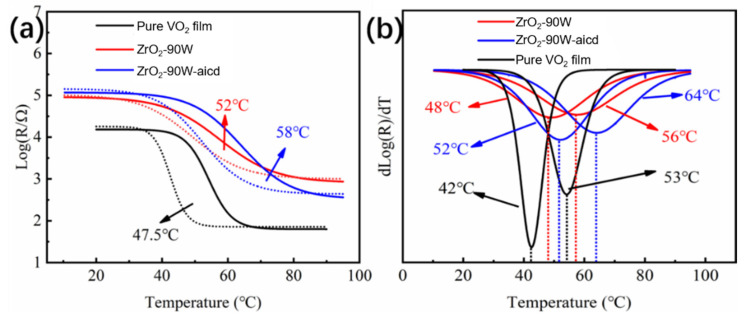
The electrical hysteresis loop (**a**) and the corresponding differential curves (**b**) of pure VO_2_ film, sample ZrO_2_-90 W, and sample ZrO_2_-90 W-acid.

As seen in Figure 7a,b, the peak of Zr 3d could be fitted into Zr 3d_5/2_ and Zr 3d_3/2_, located at 182.1 eV and 184.5 eV, respectively, corresponding to Zr^4+^. After the acid-solution process, the peaks of Zr 3d_5/2_ and Zr 3d_3/2_ were located at 182.2 eV and 184.6 eV, respectively, which also corresponded to Zr^4+^ in ZrO_2_, and the intensity of the peak was almost unchanged, indicating that the Zr content was nearly unchanged after the acid-solution process. This is probably due to the excellent chemical stability ZrO_2_ displayed in acidic solutions. After the acid-solution process, only a small amount of Zr element was probably reacted. This is confirmed by the XPS result in Figure 6. The results shows that the molar ratio of Zr/V increased from 0.02 to 0.05 during the acid-solution process, indicating that V was lost from the film at a faster rate than Zr. The 20 s of the acid-solution process did not entirely remove Zr [39]. If extending the processing time, the solar-modulation rate of the films would decrease significantly. The characteristic peak of V in the sample before the acid-solution process had two peaks corresponding to V 2p_1/2_ and V 2p_3/2_, as shown in Figure 7c. For V 2p_3/2_, there were two binding energy peaks located at 516.2 eV and 517.5 eV that could be assigned to +4 and +5 valences of V, respectively [5,40,41]. The primary source of V’s +5 valence was V_2_O_5_, indicating that the film surface was partly oxidized. O 1s spectra could be fitted into two peaks. The peak at ~530 eV was attributed to the V-O bond in the crystal lattice, whereas the peak at ~532 eV belonged to the surface-chemisorbed oxygen species [30,31]. After the acid-solution process, the film’s peak position was barely altered, whereas the content of the chemisorbed oxygen species was decreased, indicating that the island-like ZrO_2_-VO_2_ composite film exhibited good oxygen resistance, different from previous works [38]. The resistance was recorded at gradient temperatures ranging from 20 °C to 90 °C in order to determine the phase-transition temperature (*T*_c_) of samples ZrO_2_-90 W and ZrO_2_-90 W-acid. Figure 8 depicts the electrical hysteresis loop of the film. After the introduction of ZrO_2_ to the film, it can be seen that the *T*_c_ of the film increased to 52 °C, which may be attributed to the interfacial stresses caused by the ZrO_2_ and VO_2_ grains. Additionally, the electrical hysteresis loop’s breadth was about 8 °C. After the acid-solution process, some channels or voids were generated in the film and the stress between the crystal grains was released; hence, the *T*_c_ of the film rose to 58 °C and the width of the electrical hysteresis loop was expanded to 12 °C, which can be mainly attributed to the hysteresis effect in the thermal transmission process caused by the channels or voids between the grains [37,38,39].

Above all, the island-like ZrO_2_-VO_2_ composite films (ZrO_2_-90 W-acid), especially those prepared by magnetron sputtering, exhibited improved visible transmittance and superior solar modulation compared with pure VO_2_ films. As demonstrated in Table 3, the thermochromic performance attained was superior to that in the majority of earlier works.

## 4. Conclusions

In this work, ZrO_2_-VO_2_ composite films with enhanced thermochromic properties were prepared by a combination of magnetron sputtering and an acid-solution process. After the acid-solution treatment, the grains with poor crystallinity in the film were etched, and some channels or voids were generated at the same time, leading to an increase in the *T*_lum_ of the film. Without affecting the oxidation of the V film, the Δ*T*_sol_ of the film was increased with the increment of the ZrO_2_ content because its introduction could improve the crystallinity of the film. Excessive ZrO_2_ probably affected the crystallization and oxidation process of V in the film, leading to a decrease in the Δ*T*_sol_ and *T*_lum_. When the sputtering power of ZrO_2_ was 30 W and 60 W, the composite films prepared by the acid-solution process exhibited better thermochromic properties. Therefore, this work can provide a very facile and effective method to prepare VO_2_-based films with good thermochromic performance for smart windows.

## Figures and Tables

**Table 1:** Preparatory conditions of pure VO_2_ film and ZrO_2_-doped VO_2_ film.

Sample	Sputtering Pressure	Preparation of VO_2_ Thin Films		Preparation of ZrO_2_ Thin Films		Acid Treatment
		Sputtering Power	Sputtering Time	Sputtering Power	Sputtering Time	
Pure VO_2_ film	1 × 10^3^ Pa	90 W	15 min		9 times for 30 s each time	
ZrO_2_-30 W				30 W		
ZrO_2_-60 W				60 W		
ZrO_2_-90 W				90 W		
ZrO_2_-120 W				120 W		
ZrO_2_-30 W-acid				30 W		20-s
ZrO_2_-60 W-acid				60 W		
ZrO_2_-90 W-acid				90 W		
ZrO_2_-120 W-acid				120 W		

**Table 2:** The solar-modulation efficiency (Δ*T*_sol_) and visible transmittance (*T*_lum_) of the obtained films.

Sputtering Power of ZrO_2_ (W)	ZrO_2_-VO_2_ Composite Film before Acid-Solution Process		ZrO_2_-VO_2_ Composite Film after Acid-Solution Process	
	*T*_lum_ (%)	Δ*T*_sol_ (%)	*T*_lum_ (%)	Δ*T*_sol_ (%)
0	28.2	12.4	-	-
30	46.9	13.3	47.1	12.2
60	40.2	14.3	45.0	12.6
90	38.2	11.9	51.1	9.4
120	31.2	9.2	41.9	7.7

**Table 3:** Comparison of thermochromic performance of this work with previous works.

	Thermochromic Properties		Reference
System	*T*_lum_ (%)	Δ*T*_sol_ (%)	
VO_2_	39.93	9.95	Sang et al. [7]
VO_2_	55.0	18.0	Kim et al. [42]
W/Zr-doped VO_2_	60.7	10.6	Guo et al. [8]
Zn-doped VO_2_	41.3	15.3	Kang et al. [43]
Terbium-doped VO_2_ film	54.0	8.3	Wang et al. [44]
Two-dimensional nanostructure VO_2_ film	61.3	11.9	Long et al. [45]
W-doped VO_2_ film	61.7	11.7	Zhang et al. [46]
Island-like ZrO_2_-VO_2_ composite films	45.0 51.1	12.6 9.4	This work

## Data Availability

Not applicable.

## References

[1] Cui Y., Ke Y., Liu C., Chen Z., Wang N., Zhang L., Yang Z., Zhou Y., Wang S., Gao Y. (2018). Thermochromic VO_2_ for energy-efficient smart windows. Joule.

[2] Tang K., Dong K., Li J., Gordon M.P., Reichertz F.G., Kim H., Rho Y., Wang Q., Lin C., Javey A. (2021). Temperature-adaptive radiative coating for all-season household thermal regulation. Science.

[3] Wang S., Jiang T., Meng Y., Yang R., Tan G., Long Y. (2021). Scalable thermochromic smart windows with passive radiative cooling regulation. Science.

[4] Li M., Li G., Long Y. (2021). Hydrothermal Synthesis of Thermochromic VO_2_ for Energy-Efficient Windows. Vanadium Dioxide-Based Thermochromic Smart Windows.

[5] Zong H., Zhou D., Yan L., Liu H., Wu J., Hu Q., Kang C., Li M. (2021). ZrO_2_/VO_2_/ZrO_2_ sandwich structure with improved optical properties and weatherability for smart window application. Appl. Phys. A.

[6] Guo H., Wang Y.G., Fu H.R., Jain A., Chen F.G. (2020). Combined negative thermal expansion and anti-reflective effects of ZrW_2_O_8_ layer on the VO_2_ films with an enhanced luminous transmittance. Sol. Energy Mater. Sol. Cells.

[7] Sang J., Zhu W., Feng Y., Liu Y., Shang J., Sun J., Guo L., Zhang Y., Zhao S., Chigrinov V. (2021). Smart Windows with a VO_2_ Thin Film as a Conductive Layer for Efficient and Independent Dual-Band Modulation. ACS Appl. Electron. Mater..

[8] Guo H., Wang Y.G., Jain A., Fu H.R., Chen F.G. (2021). Preparation of W/Zr co-doped VO_2_ with improved microstructural and thermochromic properties. J. Alloy. Compd..

[9] Song J., Zhao Y., Sun L., Luo Q., Xu H., Wang C., Xin H., Wu W., Ma F. (2022). VO_2_/ATO nanocomposite thin films with enhanced solar modulation and high luminous transmittance for smart windows. Ceram. Int..

[10] Zou Z., Zhang Z., Xu J., Li G., Xiong R., Liu Y., Shi J. (2021). Phase transition mechanism and application of silicon-doped VO_2_ thin films to smart windows. J. Mater. Sci. Mater. Electron..

[11] Liang J., Wang S., Lei D., Wang Z., Li X. (2021). Enhanced visible and tunable infrared transmittance of W-doped VO_2_/SiO_2_/PVP composite films for smart windows. Opt. Mater..

[12] Wang C., Li N., Tu Y., Zhang J., Schmid M., Yin G. (2021). Thermochromic VO_2_ films with periodic meshes for smart windows: Analysis of optical properties. Opt. Commun..

[13] Shen N., Chen S., Shi R., Niu S., Amini A., Cheng C. (2021). Phase transition hysteresis of tungsten doped VO_2_ synergistically boosts the function of smart windows in ambient conditions. ACS Appl. Electron. Mater..

[14] Ren H., Chen S., Chen Y., Luo Z., Zhou J., Zheng X., Wang L., Li B., Zou C. (2018). Wet-Etching Induced Abnormal Phase Transition in Highly Strained VO_2_/TiO_2_ (001) Epitaxial Film. Phys. Status Solidi (RRL) Rapid Res. Lett..

[15] Savorianakis G., Mita K., Shimizu T., Konstantinidis S., Voué M., Maes B. (2021). VO_2_ nanostripe-based thin film with optimized color and solar characteristics for smart windows. J. Appl. Phys..

[16] Lv X., Chai X., Lv L., Cao Y., Zhang Y., Song L. (2021). Preparation of porous Mo-doped VO_2_ films via atomic layer deposition and post annealing. Jpn. J. Appl. Phys..

[17] Liu Y., Zhao Z.J., Li D.L., Li J.B., Zhao Y.J., Jin H.B. (2021). Infrared Switching of Self-Heating VO_2_/ITO Films for Smart Window. Trans. Tech. Publ. Ltd..

[18] Xu F., Cao X., Shao Z., Sun G., Long S., Chang T., Luo H., Jin P. (2019). Highly enhanced thermochromic performance of VO_2_ film using “movable” antireflective coatings. ACS Appl. Mater. Interfaces.

[19] Riapanitra A., Asakura Y., Yin S. (2020). One-step hydrothermal synthesis and thermochromic properties of chlorine-doped VO_2_(M) for smart window application. Funct. Mater. Lett..

[20] Liu S., Tso C.Y., Lee H.H., Du Y.W., Yu K.M., Feng S.P., Huang B. (2021). Self-Densified Optically Transparent VO_2_ Thermochromic Wood Film for Smart Windows. ACS Appl. Mater. Interfaces.

[21] Wang C., Xu H., Wang C., Liu T., Yang S., Nie Y., Guo X., Ma X., Jiang X. (2021). Preparation of VO_2_ (M) nanoparticles with exemplary optical performance from VO_2_ (B) nanobelts by ball milling. J. Alloy. Compd..

[22] Wang N., Cui Y., Gao Y., Long Y. (2021). Effect of doping on the thermochromic performance of VO_2_. Vanadium Dioxide-Based Thermochromic Smart Windows.

[23] Derkaoui I., Benkhali M., Khenfouch M., Rezzouk A. (2021). Vanadium dioxide thin films for smart windows: Numerical study and improvement of their optical properties. Newest Updates Phys. Sci. Res..

[24] Jin H., Li J. (2021). Sol-gel synthesis of thermochromic VO_2_ coatings. Vanadium Dioxide-Based Thermochromic Smart Windows.

[25] Xiao X. (2021). Antireflection for the performance of VO_2_ thermochromic thin films. Vanadium Dioxide-Based Thermochromic Smart Windows.

[26] Liu H., Zong H., Yan L., Zhou D., Yin Y., Cao G., Bian L., Kang C., Li M. (2021). SnO_2_/VO_2_/SnO_2_ tri-layer thermochromic films with high luminous transmittance, remarkable solar modulation ability and excellent hydrophobicity grown on glass substrates. Infrared Phys. Technol..

[27] Won Youn J., Lee S.J., Kim K.S., Up Kim D., Han J.W. (2021). A Study on the Coating Characteristics of VO_2_ Nanoparticle Thin Film with Various Conditions of Ultrasonic Spray Coater. J. Nanosci. Nanotechnol..

[28] Sirvent P., Pérez G., Guerrero A. (2021). Efficient VO_2_ (M) synthesis to develop thermochromic cement-based materials for smart building envelopes. Mater. Chem. Phys..

[29] Kim H.J., Choi Y.H., Lee D., Lee I.H., Choi B.K., Phark S.H., Chang Y. (2021). Enhanced passive thermal stealth properties of VO_2_ thin films via gradient W doping. Appl. Surf. Sci..

[30] Li B., Liu J., Tian S., Liu B., Yang X., Yu Z., Zhao X. (2020). VO_2_-ZnO composite films with enhanced thermochromic properties for smart windows. Ceram. Int..

[31] Li B., Yao J., Tian S., Fang Z., Wu S., Liu B., Gong X., Tao H., Zhao X. (2020). A facile one-step annealing route to prepare thermochromic W doped VO_2_ (M) particles for smart windows. Ceram. Int..

[32] Long S., Cao X., Huang R., Xu F., Li N., Huang A., Sun G., Bao S., Luo H., Jin P. (2019). Self-template synthesis of nanoporous VO_2_-based films: Localized surface plasmon resonance and enhanced optical performance for solar glazing application. ACS Appl. Mater. Interfaces.

[33] Houska J., Rezek J., Cerstvy R. (2022). Dependence of the ZrO_2_ growth on the crystal orientation: Growth simulations and magnetron sputtering. Appl. Surf. Sci..

[34] Kang L., Gao Y., Luo H., Chen Z., Du J., Zhang Z. (2011). Nanoporous thermochromic VO_2_ films with low optical constants, enhanced luminous transmittance and thermochromic properties. ACS Appl. Mater. Interfaces.

[35] Xu G., Jin P., Tazawa M., Yoshimura K. (2004). Optimization of antireflection coating for VO_2_-based energy efficient window. Sol. Energy Mater. Sol. Cells.

[36] Lee S.J., Choi D.S., Kang S.H., Yang W.S., Nahm S., Han S.H., Kim T. (2019). VO_2_/WO_3_-based hybrid smart windows with thermochromic and electrochromic properties. ACS Sustain. Chem. Eng..

[37] Li B., Tian S., Wang Z., Liu B., Gong X., Zhao X. (2021). Thermochromic Ta Doped VO_2_ Films: Enhanced Luminous Transmittance, Significantly Depressed Phase Transition Temperature and Hysteresis Width. Appl. Surf. Sci..

[38] Wang Z., Li B., Tian S., Liu B., Zhao X., Zhou X., Tang G., Pang (2021). Acid Solution Processed VO_2_-Based Composite Films with Enhanced Thermochromic Properties for Smart Windows. Materials.

[39] Zhang Y., Li B., Wang Z., Tian S., Liu B., Zhao X., Li N., Sankar G., Wang S. (2021). Facile Preparation of Zn_2_V_2_O_7_–VO_2_ Composite Films with Enhanced Thermochromic Properties for Smart Windows. ACS Appl. Electron. Mater..

[40] Zong H., Geng C., Zhang C., Liu H., Wu J., Yu Z., Cao G., Kang C., Li M. (2019). Tuning the electrical and optical properties of Zr_x_O_y_/VO_2_ thin films by controlling the stoichiometry of Zr_x_O_y_ buffer layer. Appl. Surf. Sci..

[41] Jung Y., Han H., Sharma A., Jeong J., Parkin S.S., Poon J.K. (2022). Integrated Hybrid VO_2_–Silicon Optical Memory. ACS Photon..

[42] Kim J., Paik T. (2021). Recent Advances in Fabrication of Flexible, Thermochromic Vanadium Dioxide Films for Smart Windows. Nanomaterials.

[43] Kang J., Liu J., Shi F., Dong Y., Jiang S. (2021). The thermochromic characteristics of Zn-doped VO_2_ that were prepared by the hydrothermal and post-annealing process and their polyurethane composite films. Ceram. Int..

[44] Wang N., Duchamp M., Dunin-Borkowski R., Liu S., Zeng X., Cao X., Long Y. (2016). Terbium-Doped VO_2_ Thin Films: Reduced Phase Transition Temperature and Largely Enhanced Luminous Transmittance. Langmuir.

[45] Ke Y., Wen X., Zhao D., Che R., Xiong Q., Long Y. (2018). Controllable Fabrication of Two-Dimensional Patterned VO_2_ Nanoparticle, Nanodome, and Nanonet Arrays with Tunable Temperature-Dependent Localized Surface Plasmon Resonance. ACS Nano.

[46] Zhang L., Xia F., Yao J., Zhu T., Xia H., Yang G., Liu B., Gao Y. (2020). Facile synthesis, formation mechanism and thermochromic properties of W-doped VO_2_ (M) nanoparticles for smart window applications. J. Mater. Chem. C.

